# Voyage of a Naturalist

**Published:** 1846-05

**Authors:** 


### VOYAGE OF A NATURALIST.

We recommend to all who have a taste for natural history two neat little volumes, from the press of the Harpers, under the above title; they are from the pen of Charles Darwin, M.A., F.R.S., and are among the most engaging that we have ever read. Every physician needs occasionally to turn away from his professional studies, and for the warm season which is approaching we do not know a book that would constitute a more delightful or instructive companion than the *Voyage of a Naturalist.* It abounds in details of singular interest, touching geology, botany, and every department of zoology. The publishers have brought all American fathers under obligations, by adding these volumes to the stock of our literature; eminently adapted as they are to inspire the young with a taste for reading, and for the study of natural history.

